# Tracing microbial communities associated with archaeological human samples in Latvia, 7–11th centuries AD

**DOI:** 10.1111/1758-2229.13157

**Published:** 2023-04-13

**Authors:** Jānis Ķimsis, Alise Pokšāne, Alisa Kazarina, Antonija Vilcāne, Elina Petersone‐Gordina, Pawel Zayakin, Guntis Gerhards, Renate Ranka

**Affiliations:** ^1^ Latvian Biomedical Research and Study Centre Laboratory of molecular microbiology Riga Latvia; ^2^ Institute of Latvian History University of Latvia Riga Latvia

## Abstract

In the grave environment, microorganisms are major ecological participants in the successional decomposition of vertebrates and could infiltrate the skeleton/skeletal material during taphonomic processes. The diversity of archaeological skeleton‐associated microbial assemblages and the impact of various factors are poorly understood. This study aimed to evaluate the taxonomic microbial composition of archaeological human bone and teeth samples from the 7th to 11th centuries AD from two burial sites in Latvia. Samples were analysed by a shotgun metagenomics‐based approach. The results showed a strong presence of the environmental DNA in the samples, and variability in microbial community structure between individual samples. Differences in microbial composition were observed between bone and tooth samples, as well as between different burial sites. Furthermore, the presence of endogenous ancient DNA (aDNA) in tooth samples was detected. Overall, compositions of microbial communities associated with archaeological human remains in Latvia dated 7–11th century AD were influenced by the sample type and burial location. These findings indicate that, while the content of historical DNA in archaeological samples is low, the comparison of archaeological skeleton‐associated microbial assemblages across time and space, along with aDNA damage profile analysis, is important and could help to reveal putative ancient microorganisms.

## INTRODUCTION

Next‐generation sequencing methods revolutionized the field of bioarchaeology, making it possible to execute large‐scale metagenomics studies to investigate evolutionary and ecological processes both in our ancestral societies and in their inner microbial ecosystems (Orlando & Cooper, 2014; Warinner et al., 2017). In this way, microbial archaeology explores the microbial communities associated with archaeological samples such as bones, teeth, dental calculi and anthropogenically impacted soils (Demkina et al., 2010; Margesin et al., 2017; Mulec et al., 2015; Rascovan et al., 2016; Santiago‐Rodriguez et al., 2017; Velsko et al., 2019; Xu et al., 2017). In the grave soil environment, microorganisms are major ecological participants in the successional decomposition of vertebrates (Metcalf et al., 2016), and microbial colonization of bone is an important mechanism of postmortem skeletal degradation (Emmons et al., 2020).

The vast majority of sequencing data obtained from excavated archaeological bone and tooth samples are of microbial origin, and for many ancient samples, the human endogenous DNA content is very low (<1%; Margaryan et al., 2018). Metagenome analysis of archaeological samples showed a mixture of endogenous ancient material (i.e., ancient DNA, aDNA) and exogenous sequences from colonization after the death of the organism and traces of burial environment (environmental DNA, eDNA; Kazarina et al., 2021; Warinner et al., 2017).

However, the diversity of archaeological skeleton‐associated microbial assemblages and the impact of various factors are poorly understood. This study aimed to evaluate the taxonomic microbial composition of archaeological human bone and teeth samples from the 7th to 11th centuries AD from two burial sites in Latvia.

## RESULTS

### Characteristics of archaeological samples

Archaeological human tooth and bone samples were collected from two Iron Age burials in Latvia dated 7–11th centuries AD: Lejasbiteni and Cunkani‐Drengeri, which were located near the regionally important major rivers Daugava and Mēmele, respectively (File S1).

In Cunkani‐Drengeri, individuals were buried in coarse dolomite‐containing gravel, and grave pits were filled with grey soil; in some cases, grave pits have been excavated in the dolomite base (Atgāzis, 1994). In Lejasbiteni, individuals were buried mainly in the yellow sand, less often in red clayey sand or loamy soil. Grave pits were filled with grey soil mixed with sand (Urtāns, 1964). Prior to sample collection, archaeological skeletons were inspected for possible disease signs and also age detection was performed. Characteristics of the archaeological samples including burial depth, age of individual, gender and sample type are provided in File S2.

The biological sex of adult individuals was estimated based on the morphology of the pelvis and/or skull, and the age at death was estimated based on age‐related changes in the pelvis and other skeletal elements, as well as skeletal fusion (Buikstra & Ubelaker, 1994). Sex estimation in sub‐adult individuals by macroscopic analysis of their skeletons is not possible (Lewis, 2007), thus in sub‐adult individuals, gender was estimated based on the grave orientation and grave goods (Urtāns, 1970).

### Sequencing data

All experimental procedures related to the DNA extraction from archaeological samples, sequencing library preparation, sequencing and data analysis are described in File S3. Overall, a sufficient number of reads (i.e., at least 100,000 reads) were obtained for each sample. The mean read amount per sample was 19.6 million, ranging from 13.2 million (sample LZP11.4K) to 23.7 million (sample LZP26T); the average read length ranged from 72 bp (sample LZP19.4K) to 139 bp (sample LZP13.4K; File S2). To reduce the impact of post‐mortem aDNA damage on the process of metagenomic sequence classification by KRAKEN, the reads had 5 bp trimmed from each end before the analysis.

### Taxonomic analysis: Genus level

**FIGURE 1:**
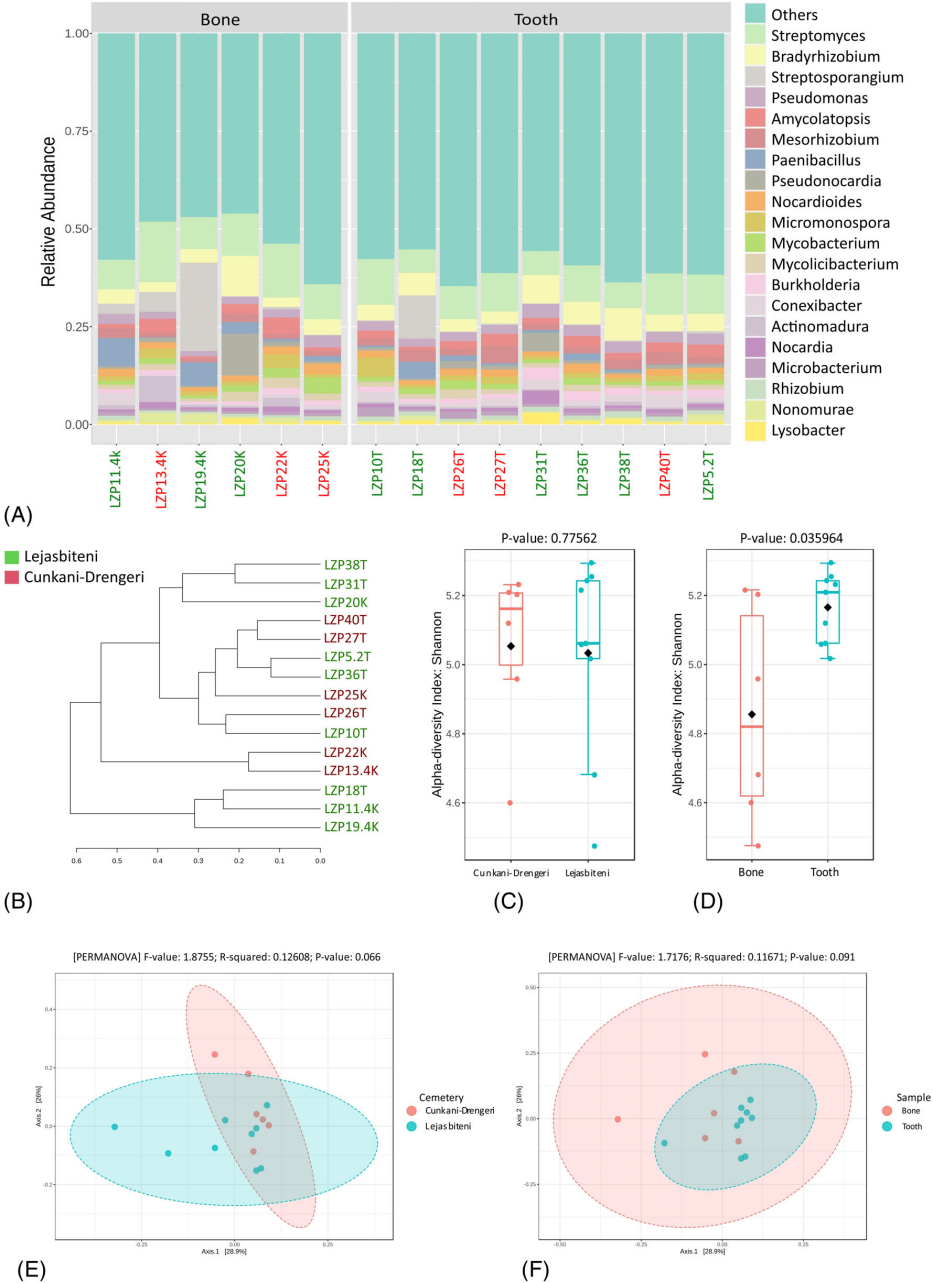
Genus‐level comparison of bacterial profiles in 7–11th century AD human archaeological tooth and bone samples: (A) Stacked plots of the taxonomic classification. The abundances of the most abundant species are shown; (B) Clustering dendrogram based on the Bray–Curtis index and Ward clustering algorithm; (C) Shannon diversity analysis for burial places; (D) Shannon diversity analysis for bone/tooth samples; (E) Permutational multivariate analysis of variance (PERMANOVA) for burial places; (F) Permutational multivariate analysis of variance (PERMANOVA) for bone/tooth samples.

Taxonomic analysis of metagenomic sequencing data of historic samples from Latvia was performed at the genus level, excluding all genera except those belonging to the domain of Bacteria, as well as the genus *Delftia*, which was detected in blank samples and is commonly associated with laboratory contamination. In total, 1618 operational taxonomic units (OTUs) were discovered within the samples and 771 OTUs were removed due to the low abundance rate (minimum mean abundance value: 100 reads). Within the remaining 847 OTUs, the most abundant genera for the bone and tooth sample pools were found to be *Streptomyces* (10.8% and 8.7%, respectively) and *Bradyrhizobium* (4.4% and 5.1%, respectively), both containing species of common soil bacteria, followed by less abundant genera (Figure 1A; File S4). A dendrogram analysis using the Bray–Curtis index and the Ward clustering method did not show a clear separation of the samples regardless of the origin/type, based on the normalized relative abundance of identified bacterial genera (Figure 1B).

While similar microbial patterns were observed between the DNA pools from different cemeteries in terms of alpha and beta diversity (*p* > 0.05; Figure 1C,E), some differences were present for the different sample types. Alpha diversity was significantly higher for the tooth samples (*p* = 0.036); however, there were no significant differences in beta diversity between the samples (Figure 1D,F).

**FIGURE 2:**
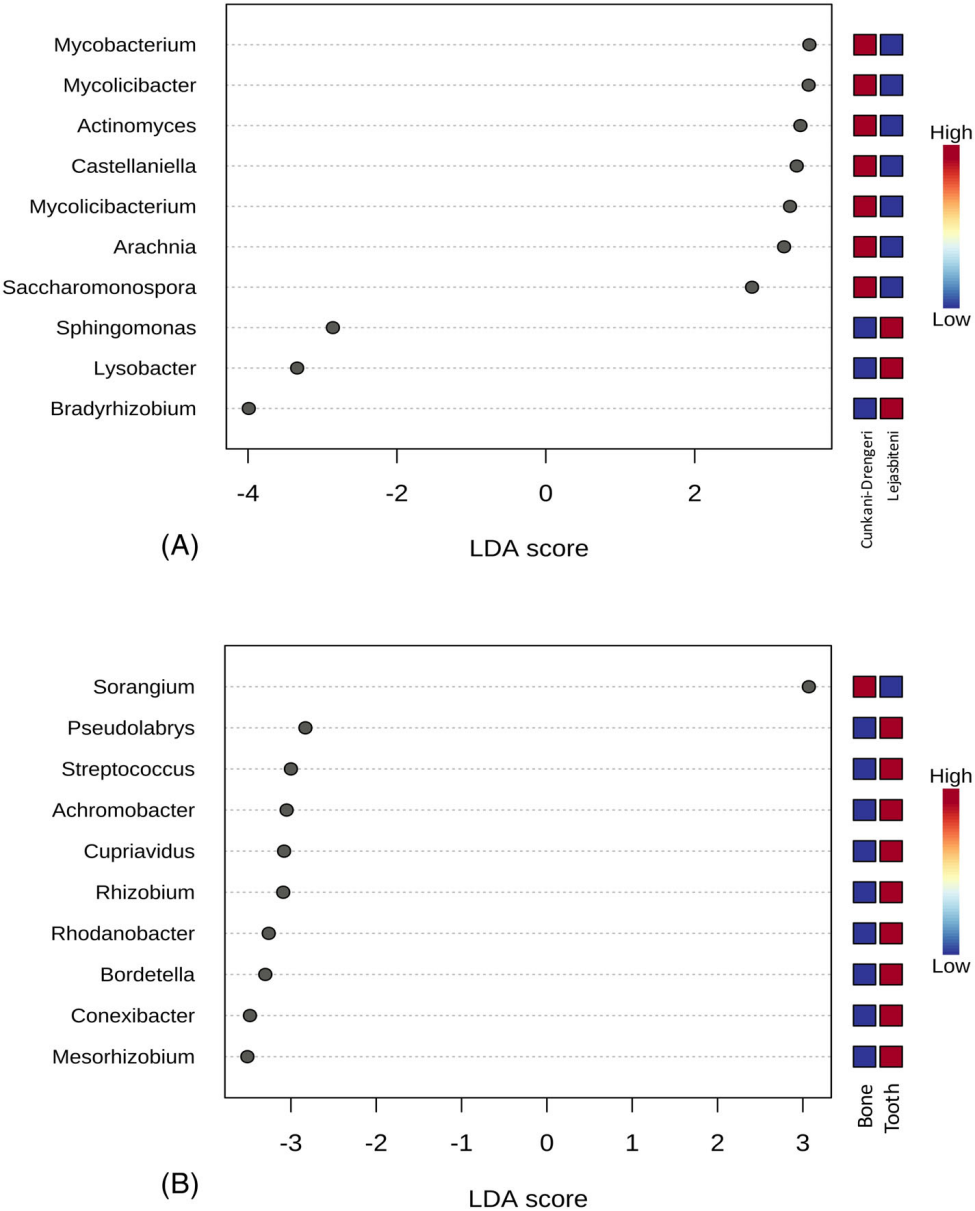
Linear discriminant analysis (LDA) combined with effect size measurements (LEfSe) identified species that enabled discrimination between the microbiotas of ancient samples, at the genus level. *p* Value cut‐off: 0.05; logarithmic LDA score ≥2.0.

Linear discriminant analysis (LDA) effect size (LEfSe) analysis with a *p* value cut‐off of 0.05 and LDA score of 2.0 found seven bacterial genera to be more prevalent in samples from Cunkani‐Drengeri (*Mycobacterium*, *Mycolicibacter*, *Actinomyces*, *Castellaniella*, *Mycolicibacterium*, *Arachnia* and *Saccharomonospora*), while three were more prevalent in the samples from Lejasbiteni (*Sphingomonas*, *Lysobacter* and *Bradyrhizobium*; Figure 2A).

When bone and tooth samples were compared, LEfSe analysis revealed that the *Sorangium* genus was more abundant in bone samples, while *Pseudolabrys*, *Streptococcus*, *Achromobacter*, *Cupriavidus*, *Rhizobium*, *Rhodanobacter*, *Bordetella*, *Conexibacter* and *Mesorhizobium* were more abundant in tooth samples (Figure 2B).

### Taxonomic analysis: Species level

**FIGURE 3:**
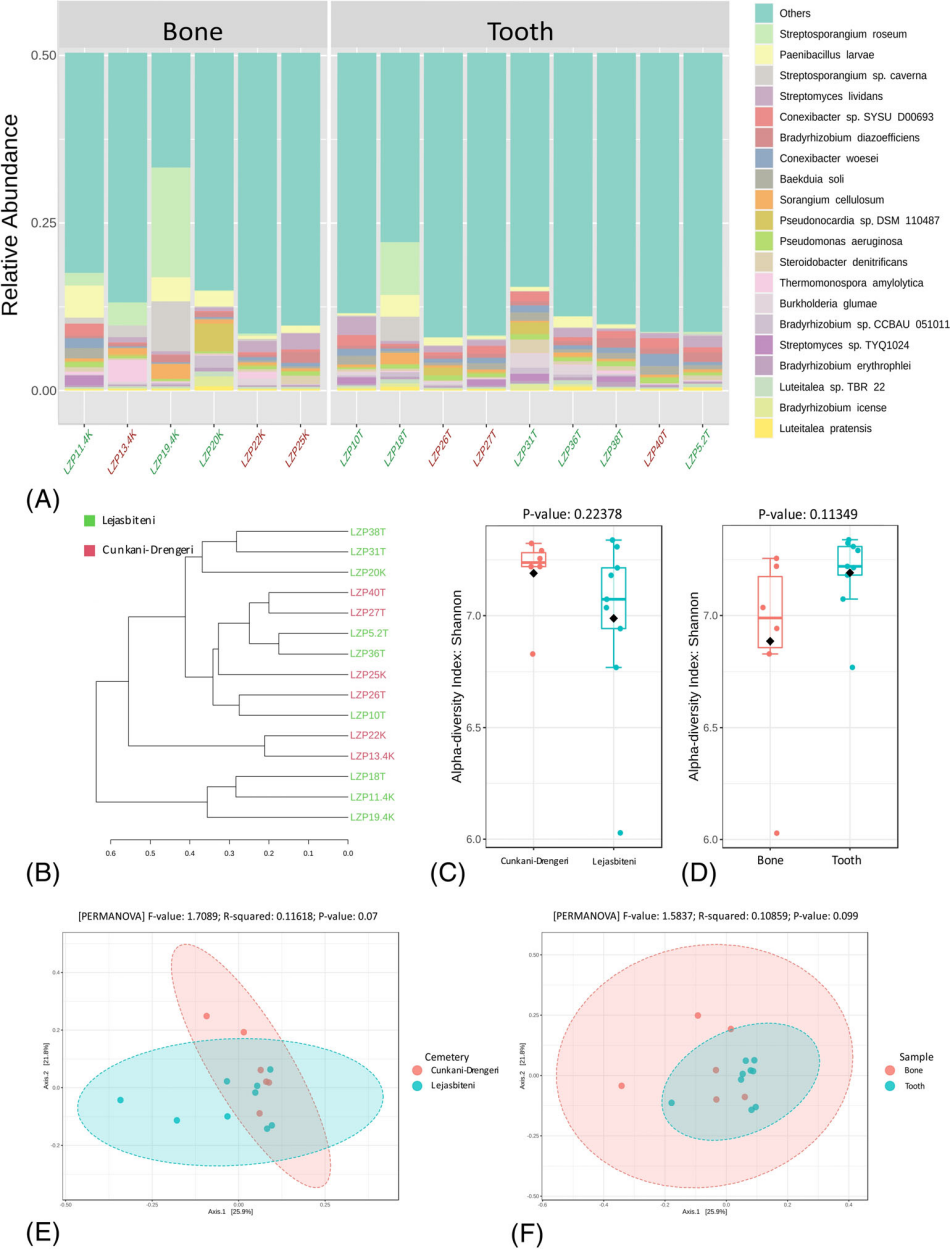
Species‐level comparison of bacterial profiles in 7–11th century human archaeological tooth and bone samples: (A) Stacked plots of the taxonomic classification. The abundances of the most abundant species are shown; (B) Clustering dendrogram based on the Bray–Curtis index and Ward clustering algorithm; (C) Shannon diversity analysis for burial places; (D) Shannon diversity analysis for bone/tooth samples; (E) Permutational multivariate analysis of variance (PERMANOVA) for burial places; (F) Permutational multivariate analysis of variance (PERMANOVA) for bone/tooth samples.

Bacterial taxa found within samples were further analysed at the species level. In total, 5892 OTUs were detected, and 2929 of them were removed due to low abundance rated (mean abundance: 200 sequences), leaving 2963 OTUs for analysis. High inter‐individual diversity between samples was observed; the most prevalent species appeared to be environmental bacteria *Streptosporangium roseum*, *Paenibacillus larvae* and *Streptosporangium sp*. ‘*caverna*’, although the amounts varied widely between different samples (Figure 3A).

**FIGURE 4:**
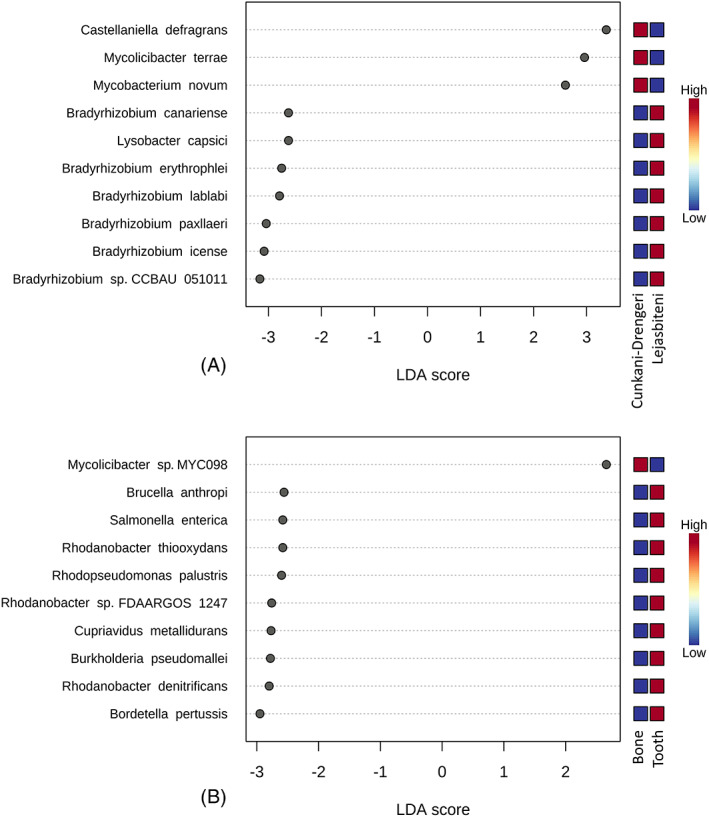
Linear discriminant analysis (LDA) combined with effect size measurements (LEfSe) identified species that enabled discrimination between the microbiotas of ancient samples, at the species level. *p* Value cut‐off: 0.05; logarithmic LDA score ≥2.0.

The dendrogram analysis did not show a clear clustering of samples by burial site or skeletal location (Figure 3B). Neither the alpha diversity (Figure 3C,D) nor beta diversity (Figure 4E,F) showed any significant differences between samples from the different burial sites or between bone/tooth samples. LEfSe analysis with a *p*‐value cut‐off of 0.05 and a high LDA score of 3.5 showed a statistically significant difference in individual species prevalence between groups.

LEfSe analysis showed a higher prevalence of *Castellaniella defragrans*, *Mycolicibacter terrae* and *Mycobacterium novum* in the samples from Cunkani‐Drengeri, while the samples from Lejasbiteni had a higher prevalence of *Bradyrhizobium canariense*, *Lysobacter capsici* and five species of *Bradyrhizobium* (*B*. *erythrophlei*, *B. lablabi*, *B. paxllaeri*, *B. icense* and *B*. *sp. CCBAU 051011*) (Figure 4A). Only one species—*Mycolicibacter sp. MYC098*—was more prevalent in the bone samples, while *Brucella anthropi*, *Salmonella enterica*, three species of *Rhodanobacter* (*R*. *thiooxydans*, *R. denitrificans* and *R*. *sp. FDAARGOS 1242*), *Rhodopseudomonas palustris*, *Cupriavidus metallidurans*, *Burkholderia pseudomallei* and *Bordetella pertussis* were more prevalent in tooth samples (Figure 4B).

### Specific aDNA damage assessment

For this analysis aimed to detect characteristic aDNA damage, which appears as cytosine (C) to thymine (T) misincorporations at the ends of aDNA fragments, untrimmed sequencing reads were used (File S3). To reduce the possibility of the alignment of sequencing reads from closely related species, sequencing reads classified to a specific species by KRAKEN were extracted using the identification numbers from the untrimmed fastq files, aligned to the appropriate reference sequence, and analysed using mapDamage software (Ginolhac et al., 2011).

The assessment of specific post‐mortem aDNA damage was performed for the 30 bacterial species with the highest relative abundance in each sample (File S5). Of those, reads of the species *Propionibacterium acidifaciens*, *Rothia aeria*, *Rothia dentocariosa*, *Streptococcus intermedius*, *Streptococcus mutans*, *Actinomyces* sp. oral taxon 169, *Actinomyces* sp. oral taxon 171 and *Actinomyces* sp. oral taxon 414 in the tooth samples LZP10T, LZP26T and LZP40T showed the characteristic aDNA damage patterns (Files S5 and S6). In addition, sequencing reads of *S. mutans* in tooth samples LZP27T and LZP10T were aDNA damage‐positive (File S4). On the contrary, in the bone samples, only sequences of *S. roseum* and *S. sp*. ‘*caverna*’ in the bone sample LZP19.4K were aDNA damage‐positive (Files S5 and S6).

## DISCUSSION

It is known that the vast majority of archaeological sample sequencing data appear to be of microbial origin (Philips et al., 2020). There is a potential possibility to uncover hidden truths about ancient human microbiome composition. On the other hand, archaeological remains‐related microbial metadata contains a portion of eDNA which could provide insight into historical environmental microbiota.

The equilibrium of the human microbiome changes quickly after death, with anaerobic bacteria persisting more successfully, and the microbial diversity of decomposing bodies starting to resemble soil microbial communities as the corpses advance to the skeletonized stage (Javan et al., 2016; Rollo et al., 2007). In our samples, the vast majority of the detected bacteria species belong to the typical soil microbiome. It was shown previously that the decomposer community was derived primarily from bulk soil, but soil type was not a dominant factor driving community development (Metcalf et al., 2016). In this study, we observed only little differences between microbial communities associated with historical human remains in two different locations, and the analysis was complicated by the variability in microbial community structure between individuals.

Soil microbiome has been investigated deeply within the last decade with many studies conducted to discover characteristic soil bacteria and their relation to macro‐environmental conditions. It has been discovered that the most common soil phyla are Proteobacteria, Actinobacteria, Acidobacteria, Verrucomicrobia, Bacteroidetes, Chloroflexi, Planctomycetes, Cytophagales, Gemmatimonadetes and Firmicutes (Buckley et al., 2003; Janssen, 2006). In our study, while burial soil samples were not available for the microbiome analysis, the 20 most prevalent bacterial genera detected in archaeological samples belonged to three phyla: Actinobacteria, Proteobacteria and Firmicutes; similar results were obtained in our recent study for bone and soil samples from the 15th to 17th century AD burials in large urban church cemeteries in Riga, Latvia (Kazarina et al., 2019). On the other hand, in contrast to this previous study, we did not observe the exceptionally high abundance of the *Firmicutes* genera in the bone samples. While the difference in the methods used—16S rRNA sequencing versus shotgun sequencing—could impact the results, this finding could also be explained by the different burial environments and the chronological age of the archaeological samples in these studies.

For the two cemeteries in the current study, we were able to identify a few differently abundant bacterial genera and species; these differences, most likely, were related to the different soil types.

For example, two bacterial species of the phylum Actinobacteria, i.e., *M. terrae* and *M. novum*, were more abundant in archaeological samples buried in dolomite‐rich soils of Cunkani‐Drengeri, while several *Bradyrhizobium* species (phylum: Proteobacteria) were more abundant in archaeological samples buried in sandy soils of Lejasbiteni. Indeed, Actinobacteria species are abundant in alkaline soils and soils rich in organic matter and can be found both on the soil surface and at depths of more than 2 m below ground (Goodfellow & Williams, 1983).

Furthermore, some notable differences in microbiome composition were observed for bone and tooth samples; importantly, traces of human microbiome including DNA sequences of the typical oral bacteria were detected in several tooth samples indicating survival of endogenous aDNA. Apart from oral microbiome trapped in dental calculus, ancient human teeth are known to be a reliable depot for ancient pathogens (Adler et al., 2011; Drancourt et al., 2007; Margaryan et al., 2018; Warinner et al., 2017). The most probable reason for this fact is the reach blood vessel network within the inner part of a tooth, allowing microorganisms to travel to this area via the bloodstream and stay trapped inside after the organism's death (Margaryan et al., 2018). In our study, the detected aDNA‐specific post‐mortem damage of the sequencing reads supports the endogenous origin of several bacterial species in the archaeological tooth samples of the 7–11th centuries AD. However, this finding could be further complicated by the fact that aDNA authentication often relies on the DNA fragment size selection, which does not eliminate the presence of historical eDNA, which tend to undergo processes of decomposition leading to a characteristic appearance of aDNA (Harrison et al., 2019; Rawlence et al., 2014). In addition, many pathogenic bacteria are members of the same genera as environmental bacteria, which can lead to false‐positive results (Campana et al., 2014).

Indeed, in our study, while the vast majority of the sequencing reads from the typical soil microbiota did not show any aDNA damage patterns, the analysis revealed post‐mortem damage for the environmental bacteria species *S. roseum* and *S. sp*. ‘*caverna*’ in one bone sample. As we were unable to find any described cases of these bacteria causing infection in humans, this finding might indicate the case of microbial invasion of the individual after interment, and thus these DNA sequences could represent ancient burial environment or ancient eDNA.

Several other possibly pathogenic bacteria species, such as *S. enterica* (Eng et al., 2015), *B. pertussis* (Nieves & Heininger, 2016), *B. anthropi* (Ryan & Pembroke, 2020), *B. pseudomallei* (Wiersinga et al., 2018) and *M. terrae* (Smith et al., 2000), were detected among the top 100 bacterial species in our samples. However, none of the reads in any sample showed the characteristic aDNA damage patterns, indicating these or closely related bacteria were present in the burial soil.

Overall, compositions of microbial communities associated with archaeological human remains in Latvia dated 7–11th century AD were influenced by the sample type and burial location. Furthermore, the likely survival of endogenous aDNA in tooth samples and historic eDNA in a bone sample was observed. These findings indicate that, while the content of historical DNA in archaeological samples is rather low, the comparison of archaeological skeleton‐associated microbial assemblages across time and space, along with aDNA damage profile analysis, is important and could help to reveal putative ancient microorganisms.

## AUTHOR CONTRIBUTIONS

**Jānis Ķimsis:** Data curation (equal); formal analysis (equal); investigation (equal); writing – original draft (equal). **Alise Pokšāne:** Data curation (equal); formal analysis (equal); investigation (equal); writing – original draft (equal). **Alisa Kazarina:** Investigation (equal). **Antonija Vilcāne:** Data curation (equal); investigation (equal). **Elina Petersone‐Gordina:** Data curation (equal); investigation (equal). **Pawel Zayakin:** Data curation (equal); software (equal). **Guntis Gerhards:** Conceptualization (equal); funding acquisition (equal); investigation (equal); methodology (equal); supervision (equal). **Renate Ranka:** Conceptualization (equal); methodology (equal); supervision (equal); writing – review and editing (equal).

## CONFLICT OF INTEREST STATEMENT

The authors declare no conflicts of interest.

## Supporting information


**Figure S1.** Location of Lejasbiteni and Cunkani‐Drengeri burial sites.

**File S2.** Characteristics of archaeological samples.

File S3.

File S4.

File S5.

File S6.

## Data Availability

Fastq files are available on European Nucleotide Archive under project number PRJEB56776.
