# Supplementary figures and images for: Tracing microbial communities associated with archaeological human samples in Latvia, 7–11th centuries AD
Source: Environ Microbiol Rep. 2023 Apr 13;15(5):383–91. doi: 10.1111/1758-2229.13157 (PMC10472514; doi:10.1111/1758-2229.13157)

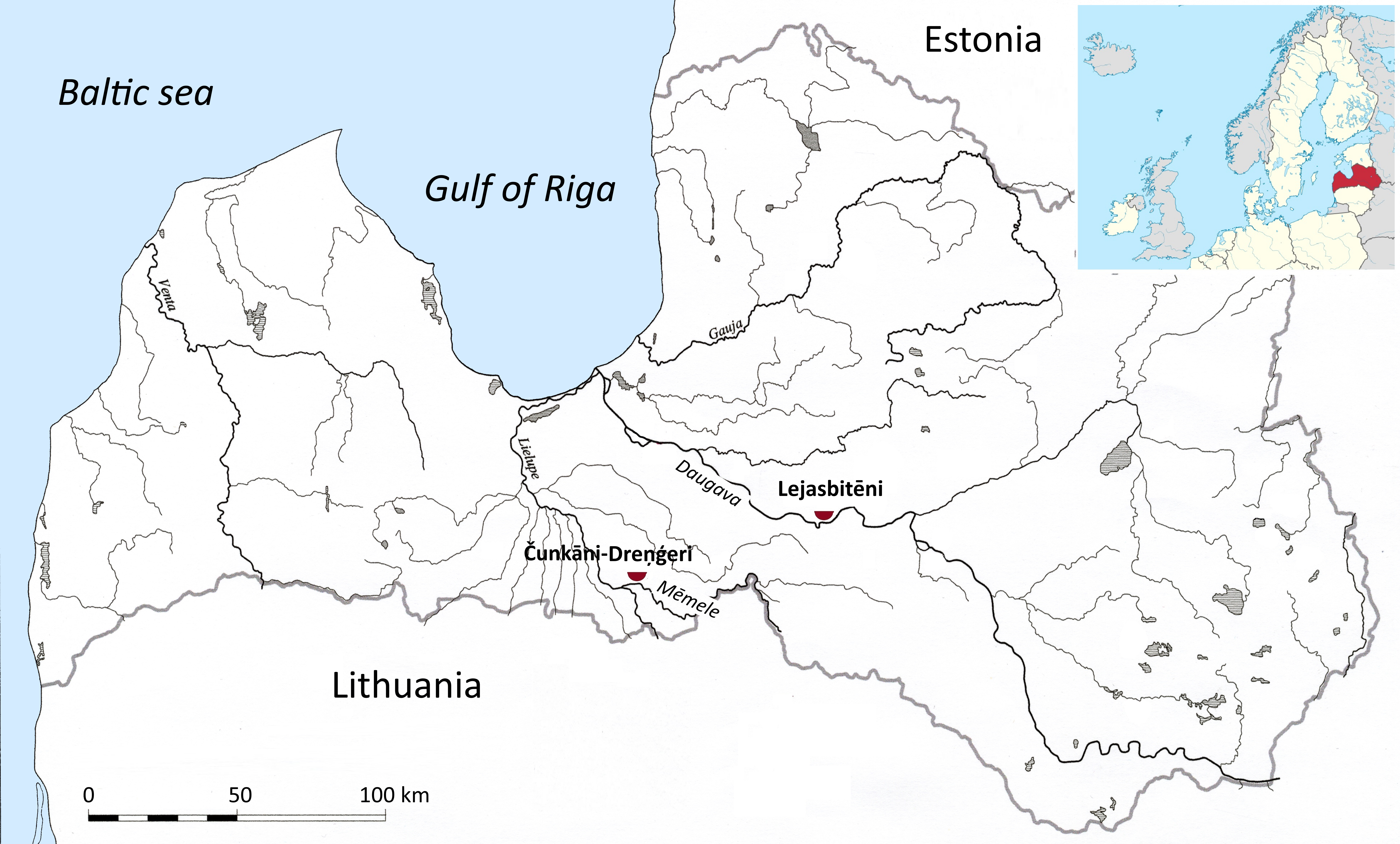

Supplement: Supplementary file 1 — Figure S1. Location of Lejasbiteni and Cunkani‐Drengeri burial sites. [file EMI4-15-383-s006.tif]

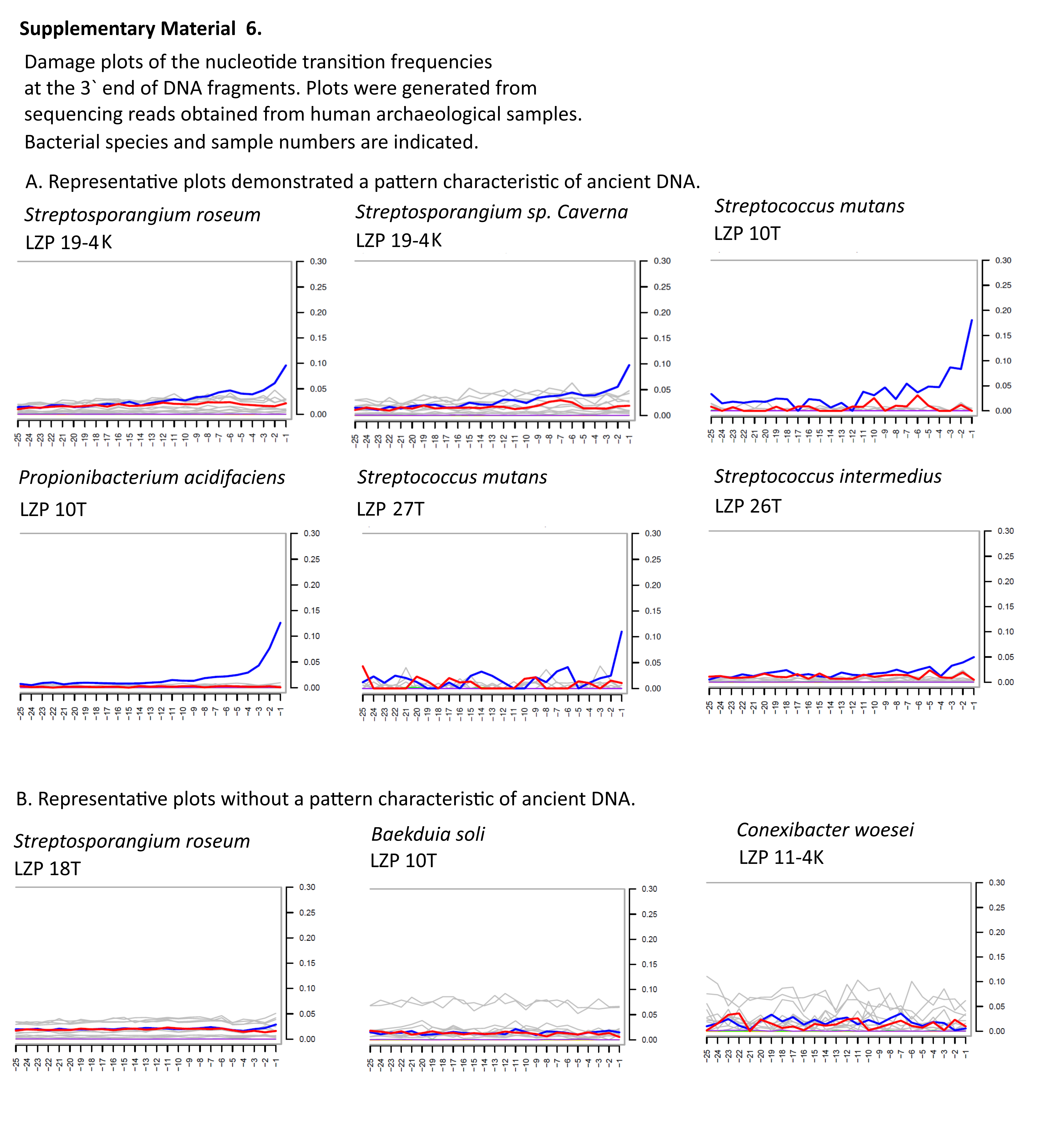

Supplement: Supplementary file 6 — File S6. [file EMI4-15-383-s001.tif]
