# Supplementary material for: Tracing microbial communities associated with archaeological human samples in Latvia, 7–11th centuries AD
Source: Environ Microbiol Rep. 2023 Apr 13;15(5):383–91. doi: 10.1111/1758-2229.13157 (PMC10472514; doi:10.1111/1758-2229.13157)
Supplement: Supplementary file 2 — File S2. Characteristics of archaeological samples. [file EMI4-15-383-s005.docx]

**Supplementary File 2.** Characteristics of archaeological samples.

| Sample number | Burial* | Grave soil type/grave pit filling | Burial depth, cm | Sex ** | Gender *** | Age of individual, years | Sample type | Bone/tooth type | Total reads obtained, mln | Bacterial reads obtained, mln (species level) | Average length of reads, bp |
| --- | --- | --- | --- | --- | --- | --- | --- | --- | --- | --- | --- |
| LZP5.2T | L | Sand/grey soil and sand | 76 | M | M | >30 | tooth | canine | 21.1 | 7.3 | 99 |
| LZP10T | L | Sand/grey soil and sand | 43 | F | F | 40-50 | tooth | molar | 18.7 | 6.7 | 112 |
| LZP11.4K | L | Sand/grey soil and sand | 55 | - | M | 8-9 | bone | petrous pyramid | 13.2 | 3.1 | 114 |
| LZP13.4K | CD | Coarse dolomite-containing gravel/grey soil | 70 | - | M | 10-12 | bone | petrous pyramid | 23.2 | 8.1 | 139 |
| LZP18T | L | Red clayey sand/grey soil and sand | 67 | - | M | 10-11 | tooth | molar | 20.9 | 7.3 | 87 |
| LZP19.4K | L | Sand/grey soil and sand | 70 | M | M | 40-50 | bone | Parietal bone | 15.8 | 7.5 | 72 |
| LZP20K | L | Loamy soil | 50 | - | M | 8-9 | bone | petrous pyramid | 23.8 | 9.4 | 111 |
| LZP22K | CD | Coarse dolomite-containing gravel/grey soil | 51 | F | F | >40 | bone | Parietal bone | 18.6 | 4.0 | 129 |
| LZP25K | CD | Coarse dolomite-containing gravel/grey soil | 50 | - | M | 7-8 | bone | petrous pyramid | 14.8 | 4.1 | 118 |
| LZP26T | CD | Coarse dolomite-containing gravel/grey soil | 45 | - | F | 12-13 | tooth | molar | 23.7 | 6.5 | 125 |
| LZP27T | CD | Coarse dolomite-containing gravel/grey soil | 70 | F | F | 20-25 | tooth | molar | 21.6 | 3.2 | 134 |
| LZP31T | L | Red clayey sand/grey soil and sand | 50 | F | F | 17-19 | tooth | molar | 21.1 | 10.7 | 115 |
| LZP36T | L | Sand/grey soil and sand | 32 | - | M | 5-6 | tooth | molar | 20.2 | 6.2 | 108 |
| LZP38T | L | Sand/grey soil and sand | 72 | - | M | 9-10 | tooth | molar | 21.0 | 8.1 | 117 |
| LZP40T | CD | Coarse dolomite-containing gravel/grey soil | 70 | M | M | 45-50 | tooth | molar | 15.9 | 7.9 | 116 |

* L – Lejasbiteni; CD – Cunkani-Drengeri

** F – female; M – male. The biological sex of adult individuals was estimated based on the morphology of the pelvis and/or skull.

*** Gender was estimated based on the grave orientation and grave goods.
