# Supplementary material for: Tracing microbial communities associated with archaeological human samples in Latvia, 7–11th centuries AD
Source: Environ Microbiol Rep. 2023 Apr 13;15(5):383–91. doi: 10.1111/1758-2229.13157 (PMC10472514; doi:10.1111/1758-2229.13157)
Supplement: Supplementary file 3 — File S3. [file EMI4-15-383-s004.docx]

**Supplementary material 3.**

**Experimental Procedures.**

*DNA extraction*

DNA extraction was performed based on the published protocol (Keyser-Tracqui and Ludes, 2005). Briefly, bone or tooth samples were washed with 10% sodium hypochlorite, 70% ethanol and deionized water to remove surface contaminants. The samples were then UV irradiated for 30 minutes from each side, covered, and left to air dry overnight. When dry, the samples were ground up in a Retsch Cryomill using one 30 second cycle of grinding. 0.5 g of the powder were transferred to a 5 ml Eppendorf tube together with 2 ml of digestion buffer containing 5 mM EDTA (PanReac AppliChem), 2% sodium dodecyl sulphate (SDS) (Fisher Bioreagents), 10 mM pH 8.0 Tris-HCl(PanReac AppliChem), 0.3M NaOAc (Sigma Aldrich) and 1 mg/ml proteinase K (ThermoFisher Scientific). One tube containing only the incubation buffer was added as a blank control, and all further operations were also carried out on the blank. The tubes were incubated for 36 hours at 50 °C with constant agitation to keep the powder suspended.

After digestion the tubes were centrifuged at 1000g for 10 minutes. The supernatant was transferred to a new 5 ml tube and 2 ml of phenol-chloroform-isoamyl alcohol (25:24:1) (Sigma Aldrich) were added, the mix was vortexed and centrifuged at 1000g for 10 minutes. The supernatant was transferred to a new 5 ml tube, 2 ml of chloroform (Acros Organics) were added, the tube was vortexed and centrifuged at 1000g for 10 minutes. After centrifugation, 1 ml of the supernatant containing the DNA was transferred to a new tube for further purification, the rest of the supernatant was stored at -20 °C.

Further, DNA was purified using the Zymo Research Genomic DNA Clean & Concentrator-10 kit. 4 ml of ChIP DNA Binding Buffer were added to the 1 ml of supernatant and mixed by pipetting. 1 ml of the mix was put into the spin column and the column was centrifuged in a tabletop centrifuge for 30 seconds. The filtrate was discarded, and the rest of the DNA mix was centrifuged through the same column, 1 ml at a time. The column was then washed by adding 200 μl of Wash Buffer and centrifuging for 1 minute. The wash was repeated for a total of three times. The column was transferred to a new Eppendorf LoBind 1.5 ml tube and aDNA was eluted by adding 30 μl of nuclease-free water, incubating at room temperature for 1 minute and centrifuging for 1 minute.

*Sequencing library preparation and sequencing*

Sequencing libraries were prepared using Qiagen Ultralow Input Library Kit for Illumina according to the manufacturer’s protocol. The quality of the libraries was checked using Agilent 2100 Bioanalyzer with High Sensitivity DNA Kit. When necessary, adapter dimers were removed using Nucleomag NGS Clean-up and Size Select Kit. The libraries were sequenced on a Illumina NextSeq 550 Series machine using a NextSeq 500/550 System Mid-Output Kit v2.5 (300 Cycles) in single end mode.

*Sequencing data analysis*

*Read pre-processing*

Primarily, sequencing data underwent initial pre-processing on local Illumina server, removing barcodes and adapters and assigning data to each sample. Further, data were exported in the format of fastq files and relocated to our local server where it was subjected to quality control protocols. Reads were trimmed using Trimmomatic (Bolger *et al.* 2014) with criteria ILLUMINACLIP:TruSeq3-SE.fa:2:30:10 SLIDINGWINDOW:4:20 to remove remaining overrepresented adapter sequences. Reads with a PHRED quality score below 20 were removed before further analysis (i.e. “Prepared reads”).

*Metagenome analysis and aDNA authentication*

The workflow of metagenome analysis and aDNA authentication is presented in Figure 1.

Step 1.

To reduce the possible impact of post-mortem aDNA damage on KRAKEN’s results, the reads had 5 bp trimmed from both ends before analysis.

Step 2.

Resultant data were analyzed by Kraken2 v2.0.7 software using databases “viral plasmid UniVec protozoa fungi human archaea bacteria” in order to assign taxonomic labels to the resultant metagenomics sequences (Wood and Salzberg, 2014). Kraken taxonomy was extracted in the form of report files which were further manipulated with the use of Pavian web application; taxa other than Bacteria were removed in order to generate taxonomy quality assignment (Breitwieser and Salzberg, 2020). Microbial genera and species, which were detected in the blank controls (the common laboratory bacteria genus *Delftia*) were removed from the sample datasets.

Step 3.

To check for damage patterns characteristic of aDNA, the “prepared reads” were used, i.e. sequencing reads that were processed but did not have 5 bp trimmed from both ends.

To filter out these reads, first, the IDs of sequencing reads which were classified to the top 30 most abundant bacterial species were extracted from Kraken .krkn files using the bash command
awk -v a=$id '$3==a {print $2}' $infile > $outfile, where $id is the taxonomic ID of the species, $infile is the .krkn file and $outfile is the file to write the IDs to.

Step 4

Next, sequencing reads belonging to a specific species were extracted from the prepared reads based on their IDs using using the seqtk1.3 tool (Li, 2013), command subseq.

Step 5

Extracted reads were aligned to that species reference sequence with bwa mem using default options. Reads with mapping quality below 30 were removed with samtools view (Danecek *et al*. 2021) and reads that had any softclipping (i.e., where the ends of the reads did not align to the reference sequence) were removed with samclip (Seemann 2020) using option –max 0.

Step 6. The remaining reads were analyzed using MapDamage v2.2.1 (Ginolhac et al. 2011).


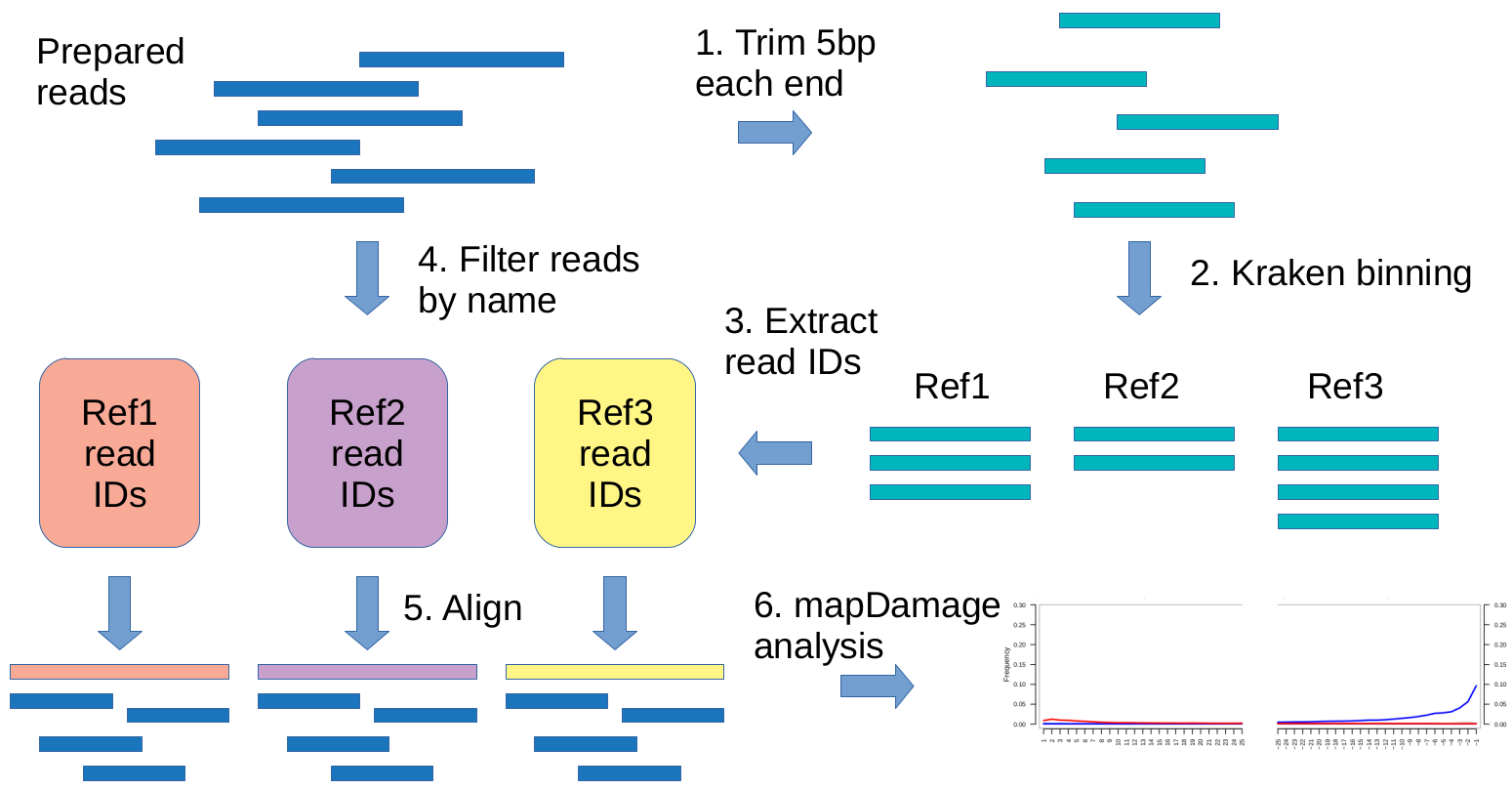

Figure 1. Extraction of reads based on KRAKEN binning results.

*Evenness of coverage*

To check for evenness of coverage, the number of aligned reads for the 20 most prevalent species for each sample were plotted against the coverage for the given sample, which showed a linear trend (Figure 2).


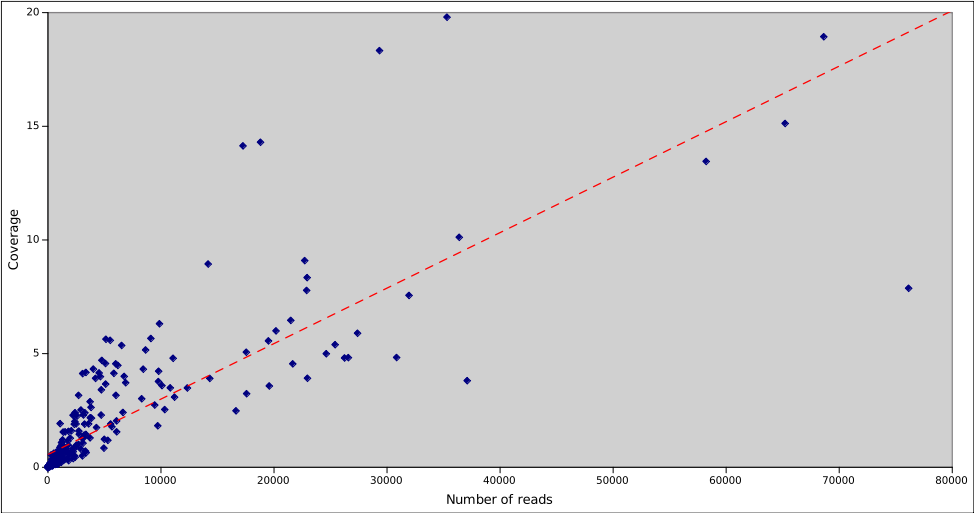


Figure 2. Evenness of coverage for 20 most common bacterial species in all samples analysed (N = 15).

*Microbiome analysis*

MicrobiomeAnalyst public server (<https://www.microbiomeanalyst.ca/>) was used to perform statistical analyses and result data visualizations (Dhariwal et al., 2017; Chong et al., 2020).

For this analysis, Kraken results from trimmed sequencing reads (Step 2) were used.

Low-abundance species were removed with the criteria minimum count 100 and mean abundance value, and all samples were rarefied to even sequencing depth based on the sample having the lowest sequencing depth. Alpha diversity of microbial communities was estimated using Mann-Whitney/Kruskal-Wallis statistic and Shannon diversity index, and permutational multivariate analysis of variance (PERMANOVA) and a Bray-Curtis dissimilarity matrix were used to assess differences in beta diversity between sample clusters. Principal component analysis (PCA) was further used to visualize the results.

Hierarchical clustering was visualized by dendrogram and heatmap analysis using the Bray–Curtis similarity index and Ward clustering algorithm. Intergroup differences at the genus and species level were analyzed using the Kruskal–Wallis test and the linear discriminant analysis (LDA) effect size (LEfSe) method (Segata et al., 2011) with default settings on the MicrobiomeAnalyst website; the threshold on the logarithmic LDA score for discriminative features was set to 2, and the p value cutoff was set to 0.05.

**References**

1. Atgāzis, M. (1994) Dreņģeru-Čunkānu kapulauks un zemgaļu senvēstures pētniecības jautājumi . Zinātniskās atskaites sesijas materiāli par arheologu 1992. un 1993. gada pētījumu rezultātiem. (Drengeri-Cunkani burial ground and questions of ancient Semigallian research. Materials of the scientific report session on the results of the 1992 and 1993 researches of archaeologists). Rīga; Zinātne, 23-30. In Latvian.

2. Bolger, A.M., Lohse, M., Usadel, B. (2014). Trimmomatic: a flexible trimmer for Illumina sequence data. – Bioinformatics 30, 15: 2114–2120.

3. Breitwieser, F.P., Salzberg, S.L. (2020) Pavian: interactive analysis of metagenomics data for microbiome studies and pathogen identification. Bioinforma Oxf Engl 36:1303–4.

4. Chong, J., Liu, P., Zhou, G., Xia, J. (2020) Using MicrobiomeAnalyst for comprehensive statistical, functional, and meta-analysis of microbiome data. Nat Protoc 15:799–821.

5. Danecek, P., Bonfield, J. K., Liddle, J., Marshall, J., Ohan, V., Pollard, M. O., Whitwham, A., Keane, T., McCarthy, S. A., Davies, R. M., Li H. (2021). Twelve years of SAMtools and BCFtools. GigaScience 10, 2: giab008.

6. Dhariwal, A., Chong, J., Habib, S., King, I.L., Agellon, L.B., Xia, J. (2017) MicrobiomeAnalyst: a web-based tool for comprehensive statistical, visual and meta-analysis of microbiome data. Nucleic Acids Res 45:W180–8.

7. Ginolhac A., Rasmussen M., Gilbert M. T. P., Willerslev E., Orlando L. (2011) mapDamage: testing for damage patterns in ancient DNA sequences. – Bioinformatics 27, 15: 2153–2155.

8. Keyser-Tracqui, C., Ludes, B. (2005) Methods for the Study of Ancient DNA. In: Carracedo A, editor. Forensic DNA Typing Protoc [Internet]. Totowa, NJ: Humana Press, p. 253–64. Available from: https://doi.org/10.1385/1-59259-867-6:253

9. Li, H. (2013). Aligning sequence reads, clone sequences and assembly contigs with BWA-MEM. <https://arxiv.org/abs/1303.3997>

10. Li, H. (2013). Seqtk: a fast and lightweight tool for processing FASTA or FASTQ sequences. <https://github.com/lh3/seqtk>. Last accessed 2022.09.06.

11. Seemann, T. (2020). Samclip. https://github.com/tseemann/samclip. Last accessed 2022.09.06.

12. Segata, N., Izard, J., Waldron, L., Gevers, D., Miropolsky, L., Garrett, W.S., Huttenhower, C. (2011) Metagenomic biomarker discovery and explanation. Genome Biol 12, R60. doi:10.1186/gb-2011-12-6-r60.

13. Urtāns, V. (1961) Pārskats par arheoloģiskajiem izrakumiem Aizkraukles Lejasbitēnu kapulaukā 1961. - 1964. gadā (Overview of archaeological excavations in the Aizkraukle Lejasbitēni cemetery in 1961 – 1964) Archaeological report in NHML, Rīga. In Latvian.

14. Wood, D.E., Salzberg, S.L. (2014) Kraken: ultrafast metagenomic sequence classification using exact alignments. Genome Biol 15:R46.
